# A comprehensive assessment of lymphocyte subsets, their prognostic significance, and changes after first‐line therapy administration in patients with chronic lymphocytic leukemia

**DOI:** 10.1002/cam4.5492

**Published:** 2022-11-28

**Authors:** Pavel Vodárek, Dominika Écsiová, Vladimíra Řezáčová, Ondřej Souček, Martin Šimkovič, Doris Vokurková, David Belada, Pavel Žák, Lukáš Smolej

**Affiliations:** ^1^ 4th Department of Internal Medicine – Hematology Faculty of Medicine University Hospital and Charles University Hradec Kralove Czech Republic; ^2^ Institute of Clinical Immunology and Allergology Faculty of Medicine University Hospital and Charles University Hradec Kralove Czech Republic

**Keywords:** chemoimmunotherapy, CLL, immunosuppression, infections, lymphocyte subset, prognosis

## Abstract

**Background:**

In chronic lymphocytic leukemia (CLL), changes in the peripheral blood lymphocyte subsets play an important role in disease progression and infectious complications. The impact of chemoimmunotherapy (CIT) on these changes has not been extensively studied

**Methods:**

We used multi‐color flow cytometry, to prospectively measure absolute and relative numbers of CD4^+^ and CD8^+^ T‐cells and their subsets in 45 patients with indolent untreated CLL, 86 patients indicated for first‐line treatment, and 34 healthy controls. In 55 patients, we analyzed the impact of CIT

**Results:**

CLL patients had a significant increase in most cell populations in comparison to controls. Progression of CLL was characterized by significantly elevated counts with the exception of a lower percentage of naïve T‐cells. After treatment, the percentage of naïve T‐cells further decreased at the expense of effector memory T‐cells (TEM). In patients with indolent CLL, higher percentages of naïve CD4^+^ (*p* = 0.0026) and naïve CD8^+^ (*p* = 0.023) T‐cells were associated with a longer time to first treatment (TTFT). The elevation of CD4^+^ central memory T‐cells (TCM) (*p* = 0.27) and TEM (*p* = 0.003) counts and a higher percentage of CD4^+^ TEM (*p* = 0.0047), were linked with shorter TTFT. In treated patients, increased regulatory T‐cells count was associated with shorter time to next treatment (TTNT) (*p* = 0.042), while higher CD4^+^ TCM count with shorter TTNT (*p* = 0.035) and shorter overall survival (*p* = 0.041).

**Conclusion:**

Our results indicate that naïve cell depletion and CD4^+^ TCM and TEM increases are detrimental to CLL patients' prognosis.

## Graphical abstract

Multi‐color flow cytometry analysis of absolute and relative numbers of CD4^+^ and CD8^+^ T‐cells and their subsets in patients with both indolent and progressive chronic lymphocytic leukemia. The effect of chemoimmunotherapy is investigated and evidence for the negative prognostic impact of naïve cells depletion and central/effector memory cell increase is presented.

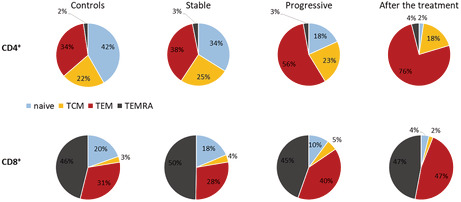

## 1 INTRODUCTION

Chronic lymphocytic leukemia (CLL) is characterized by complex alterations of lymphocyte counts and functions.^1^, ^2^, ^3^, ^4^, ^5^, ^6^, ^7^, ^8^, ^9^, ^10^, ^11^, ^12^, ^13^, ^14^ The resulting immune system dysfunctions contribute to disease progression and a higher susceptibility to infections which is the most important cause of morbidity and mortality in CLL patients.^15^ Physiologically, CD4^+^ and CD8^+^ T lymphocytes undergo development from naïve T‐cells to central memory T‐cells (TCM), that further differentiate into effector memory T‐cells (TEM) and finally on to terminally differentiated effector memory T‐cells (TEMRA).^16^, ^17^

Some authors observed a shorter time to first treatment (TTFT) in patients with a lower ratio of total T‐cells and natural killer (NK) cells to clonal B lymphocytes.^1^ Others described an association of shorter TTFT, progression‐free survival (PFS), or overall survival (OS) with an inversion of the CD4^+^ to CD8^+^ ratio.^2^, ^3^ The expansion in the TEM and TEMRA subsets were associated with the disease stage, unmutated immunoglobulin heavy chain variable region (IGHV) genes and deletions in chromosomes 11q and 17p.^4^, ^5^ A number of studies which focused on regulatory T‐cells (T‐reg) revealed an increase of either their relative number or absolute count in comparison to healthy controls.^6^, ^7^, ^8^, ^9^, ^10^ Many of these studies established connections between the T‐reg number and the stage of the disease and even TTFT.^10^, ^11^, ^12^ NK cells are also increased in CLL, but contrary to T‐reg, they may play more of a protective role, as their lower count was connected to shorter OS.^13^ Natural killer T (NKT) cells are a heterogeneous subset of T‐cells that are capable of both regulatory (production of cytokines) and effector (lysis of target cells) functions. Their percentage is significantly reduced in patients with progressive disease compared to those with stable disease or healthy controls.^14^

Chemoimmunotherapy (CIT) has long been the standard of care in the treatment of CLL. Although it is being phased out in favor of newer targeted therapies (venetoclax, Bruton tyrosine kinase inhibitors), it still remains a viable option for untreated patients with mutated IGHV without TP53 dysfunction, especially in countries with significant limitations in their healthcare budgets.^18^ CIT can aggravate disease‐related immunosuppression with a decrease in lymphocyte counts, resulting in a higher frequency of infections including opportunistic ones. FCR (fludarabine, cyclophosphamide, rituximab) treatment causes a depletion of CD4^+^ cells and also to a lesser extent, CD8^+^ and NK cells.^19^, ^20^, ^21^ There is sufficient data on the lymphocyte count decrease after BR (bendamustine, rituximab) therapy, however, most of the published works do not specifically focus on CLL.^22^, ^23^, ^24^, ^25^ Currently, there is no data available on the effect of BR on the more specific lymphocyte subsets or T‐reg numbers and only two reports have been published about the effect of chlorambucil on the lymphocyte populations.^26^, ^27^

### 1.1 Aims of the study

To analyze lymphocyte subsets in patients with stable CLL and progressive CLL before as well as after first‐line treatment with CIT and to examine for any possible associations between the lymphocyte subsets, prognosis of CLL, and development of infectious complications.

## 2 PATIENTS AND METHODS

### 2.1 Data acquisition

This was a single‐center prospective observational study. Patients with CLL diagnosed according to the IWCLL criteria of 2008, followed and treated at the 4th Department of Internal Medicine—Hematology, University Hospital in Hradec Králové, Czech Republic, were prospectively evaluated from September 2013 until November 2020. The referral region of the center covers parts of Eastern Bohemia with a population of about 1 million inhabitants. All of the patients who agreed to participate in the study signed the necessary informed consent forms and the study was approved by the local ethics committee and conducted according to the principles of the declaration of Helsinki. Peripheral blood samples were taken upon entry into the study in patients with the indolent disease and before and after treatment (2–3 months after the last cycle of CIT) in patients indicated for the first‐line treatment. There were no specific exclusion criteria. Patients with progressive disease were indicated for treatment based on the IWCLL criteria. The dosing of the FCR, BR, and O‐Clb (obinutuzumab, chlorambucil) regimens corresponded to those used in CLL10 and CLL11 studies.^28^, ^29^ The dosing of R‐Clb (rituximab, chlorambucil) was the same as in the CLL 208 study.^30^ We collected the following data on the patient's disease course: the Rai stage at the time of blood collection, the development of any infectious complications, TTFT and OS in patients with stable disease, time to next treatment (TTNT), and OS of the patients with progressive disease who were indicated for treatment. We defined OS, TTFT, and TTNT as the time from blood collection, rather than the time from diagnosis because the blood collection was not performed at the time of diagnosis in a significant proportion of patients. The blood samples for the control group were obtained from healthy blood donors.

### 2.2 Flow cytometry analysis

We measured the absolute and relative numbers of total T‐cells, CD4^+^ T‐cells, CD8^+^ T‐cells, CD4^+^CD8^+^ T‐cells, CD4^−^CD8^−^ T‐cells, total, clonal, and polyclonal B‐cells, NKT and NK cells and T‐reg. In addition, we measured major functional subsets of both CD4^+^ and CD8^+^ T‐cells (naïve, TCM, TEM, and TEMRA). The staining processes along with the detailed descriptions of the procedures used in the analysis are provided in Appendix S1 The gating strategies for the lymphocyte subsets that were assessed were as follows: lymphocytes (low side scatter/CD45^++^), T‐cells (CD3^+^), NK cells (CD3^−^CD16^+^ and/or CD56^+^), NKT cells (CD3^+^CD56^+^), helper T‐cells (CD3^+^CD4^+^), cytotoxic T‐cells (CD3^+^CD8^+^), B‐cells (CD19^+^), clonal B‐cells (CD19^+^, CD5^+^, CD20low, restriction kappa/lambda), polyclonal B‐cells (CD19^+^, CD20^+^, normal ratio kappa/lambda), naïve helper T‐cells (CD3^+^CD4^+^CD45RA^+^CD197^+^), helper TCM (CD3^+^CD4^+^CD45RA^−^CD197^+^), helper TEM (CD3^+^CD4^+^CD45RA^−^CD197^−^), helper TEMRA (CD3^+^CD4^+^CD45RA^+^CD197^−^), naïve cytotoxic T‐cells (CD3^+^CD8^+^CD45RA^+^CD197^+^), cytotoxic TCM (CD3^+^CD8^+^CD45RA^−^CD197^+^), cytotoxic TEM (CD3^+^CD8^+^CD45RA^−^CD197^−^), cytotoxic TEMRA (CD3^+^CD8^+^CD45RA^+^CD197^−^), and the T‐reg (CD3^+^CD4^+^CD25^++^CD127^−^FoxP3^+^).

Relative numbers of total T‐cells, clonal and polyclonal B‐cells, and NK cells are given as a percentage of all lymphocytes in which their sum is equal to 100%. Relative numbers of CD4^+^ T‐cells, CD8^+^ T‐cells, CD4^+^CD8^+^ T‐cells, CD4^−^CD8^−^ T‐cells, and NKT cells are also given as a percentage of all lymphocytes and the sum of their percentages is equal to that of total T‐cells. Relative numbers of naïve, TCM, TEM, and TEMRA cells are given as a percentage of all CD4^+^ or CD8^+^ T‐cells. The relative number of T‐reg is a percentage of CD4^+^ T‐cells. The relative numbers of cells obtained via flow cytometry analysis were multiplied by the leukocyte count which was obtained from the routine blood count in order to calculate the absolute numbers of cells.

### 2.3 Statistics

All of the statistical analyses were performed by using MedCalc software, version 20 (MedCalc Software Ltd). The Shapiro–Wilk test was used to assess the data distribution of the samples. In cases where both compared data sets had normal distributions of data, the independent samples *t*‐test was performed to compare the different cohorts and the paired samples *t*‐test to determine the statistically significant changes in the longitudinal observations (patients before and after the treatment). In case of any abnormal data distributions, the Mann–Whitney *U* test was used for the independent samples and the Wilcoxon test, for the paired samples. The Cox‐proportional‐hazards regression method was used in determining the impact of different lymphocyte subset counts on TTFT, TTNT, and OS. The variables identified as being statistically significant in the univariate analysis were included in the multivariate analysis. The analysis of the receiver operating characteristics (ROC) curve, was performed to establish the ideal cut‐off level for the best separation of the survival curves, which were constructed according to the Kaplan–Meier method and compared using the log‐rank test. In all cases, the *p*‐values are double‐sided and the values ≤0.05, are considered significant.

## 3 RESULTS

**FIGURE 1:**
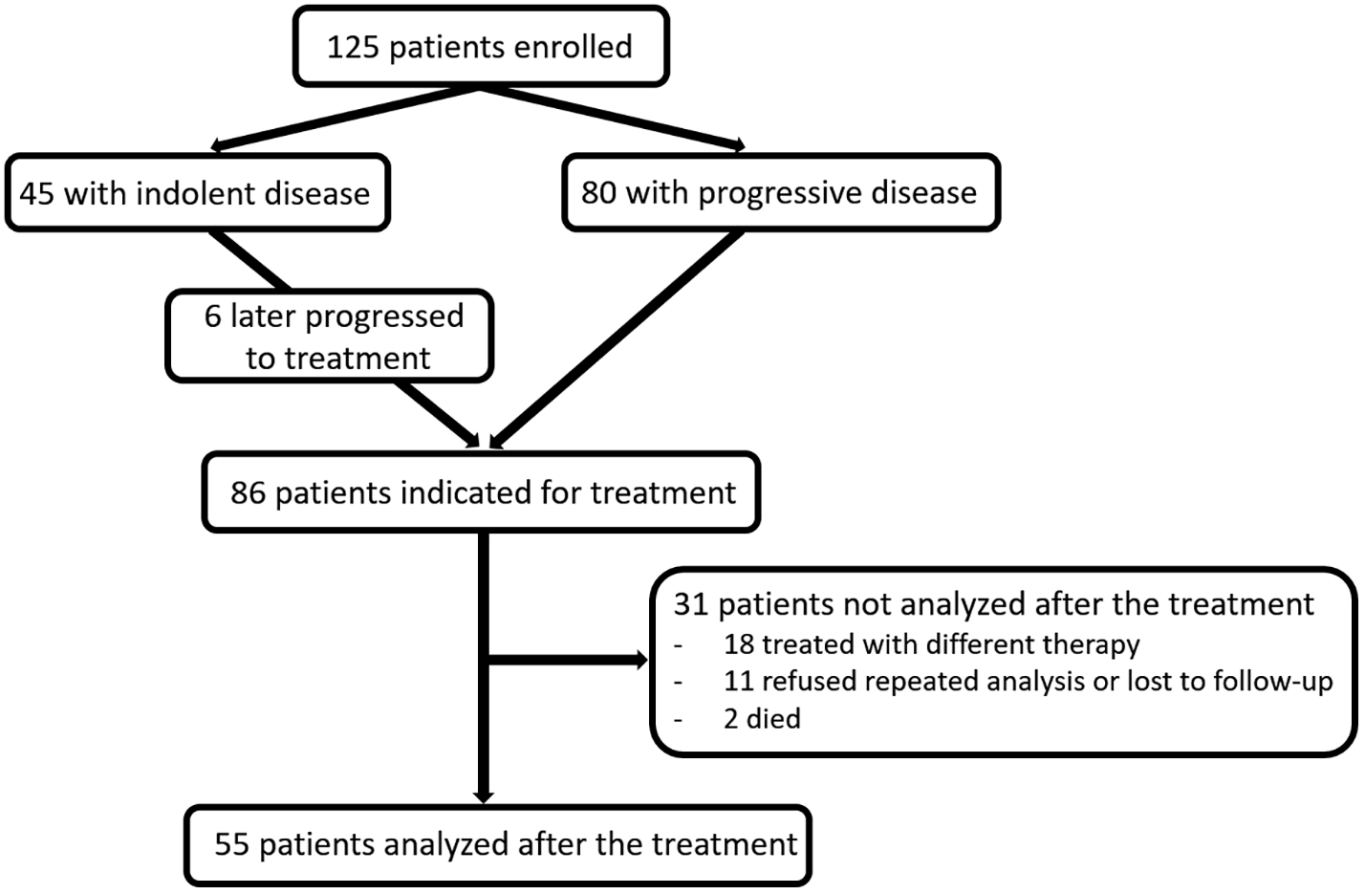
Flowchart representing patients’ disposition

Between September 2013 and May 2019, we enrolled 125 patients with CLL who were followed up until November 2020. Their basic characteristics are summarized in Table 1 and Table S1. A total of 34 healthy blood donors were used as the control group (median age 60 years [range, 43–67], 56% males). There were 45 patients with stable disease (without any indication for treatment) and 86 patients with progressive disease who were indicated for treatment with six of them having been analyzed before at the time of diagnosis as part of the stable cohort. These six were only used for comparison with controls. Out of the 86 patients who were indicated for treatment, 18 were not analyzed after receiving treatment because they were treated with regimens other than FCR, BR, R‐Clb, or O‐Clb. The wide variations of these other treatment modalities would complicate the statistical analysis. Furthermore, for various reasons, the blood samples after therapy were not available in 13 patients. Therefore, a repeated analysis after the treatment was done in 55 patients (Figure 1). Therapy using FCR/BR/chlorambucil‐based regimens was used in 17, 18, and 20 patients, respectively. The response to treatment (partial or complete remission) was achieved in 17/17 patients after FCR, 16/18 after BR, 8/11 after R‐Clb, and 8/9 after O‐Clb.

**TABLE 1:** Characteristics of the patient cohorts

Characteristic	Stable	Progressive	After the treatment
Total number	45	86	55
Age, median (range) at the date of blood collection	65 years (34–88)	68 years (43–87)	68 years (43–87)
Males (%)	24 (53)	57 (66)	32 (58)
Median follow‐up from the date of blood collection (months)	49	39	29
Median time (range) from diagnosis to the date of blood collection	26 months (4–141)	34 months (0–174)	42 months (1–174)
Rai modified risk at the date of blood collection (%)			
Low	27 (60)	1 (1)	1 (2)
Intermediate	16 (36)	33 (38)	22 (40)
High	2 (4)	52 (60)	32 (58)
IGHV (%)			
Mutated	25 (56)	19 (22)	11 (20)
Unmutated	10 (22)	55 (64)	34 (62)
NA	10 (22)	12 (14)	10 (18)
*TP53* (%)			
Mutated	0	9 (10)	5 (9)
Unmutated	30 (67)	63 (73)	39 (71)
NA	15 (33)	14 (16)	11 (20)
FISH (%)			
Normal	9 (20)	17 (20)	15 (27)
13q deletion	24 (53)	25 (29)	17 (31)
12 trisomy	4 (9)	15 (17)	8 (15)
11q deletion	2 (4)	15 (17)	8 (15)
17p deletion	0	9 (10)	2 (4)
NA	6 (13)	5 (6)	5 (9)
*TP53* mutation or 17p deletion	0	13 (15)	6 (11)

*Note*: Data for the “After the treatment,” cohort correspond to the pre‐treatment values. Rai modified risk: stage 0 = low, stage I/II = intermediate, stage III/IV = high. IGHV–mutational status of the immunoglobulin heavy chain variable region. FISH–cytogenetic aberrations detected by fluorescence in situ hybridization. *TP53*–mutation of tumor protein 53 investigated by Sanger sequencing. NA–not available.

### 3.1 Cell subset ratios

The median CD4^+^: CD8^+^ ratio of the control group was 1.99 (range, 1.31–3.01), which was not significantly different from that of the stable patient's group (*p* = 0.48) or the progressive disease group (*p* = 0.13) (Table S2). There was also no significant difference between patients with stable and progressive disease (*p* = 0.64). After treatment, the median CD4^+^:CD8^+^ ratio decreased to 0.73 (range 0.17–8.63). This is significantly lower than that of controls, stable patients, and patients before CIT administration (*p* < 0.0001 for all comparisons), which reflected a comparatively more pronounced decrease in CD4^+^ cells after treatment.

We also analyzed the ratios of absolute counts of CD4^+^, CD8^+^, all T‐cells, NK, and NKT cells to clonal B‐cells. Only a comparison between the patients with the stable and progressive disease was done in this regard due to a significant depletion of clonal B‐cells in the patients after therapy. Not surprisingly, all of these ratios were significantly lower in the patients with progressive disease due to higher clonal B‐cell counts.

The possible relationship of all of the mentioned ratios to TTFT in stable patients and OS and TTNT in patients with progressive disease were investigated. OS was not analyzed in the stable patients' group because of only three deaths during the follow‐up period. The lower ratio of NKT to clonal B‐cells in patients with progressive disease was associated with a shorter OS (*p* = 0.043). The other ratios had no relationship to any of the analyzed survival times.

### 3.2 Changes in the different subset counts

**FIGURE 2:**
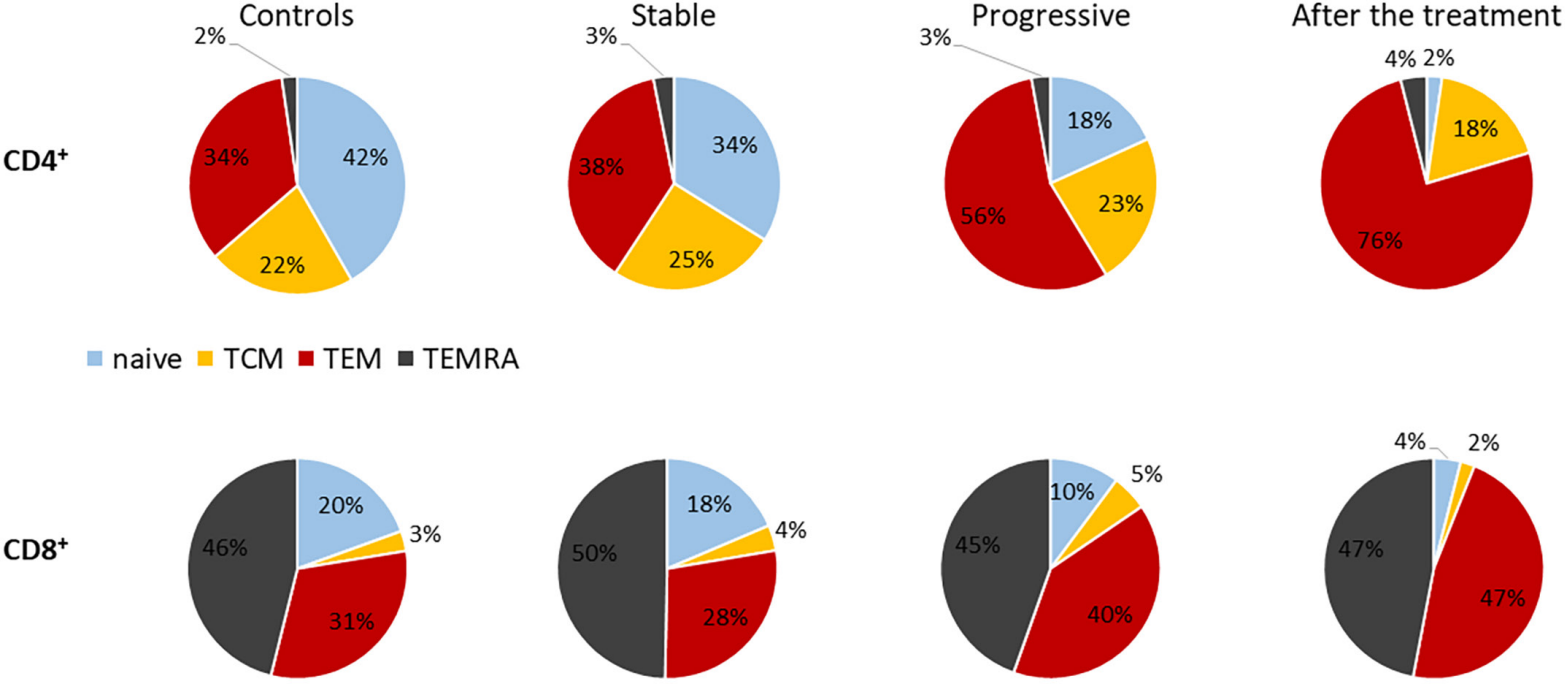
Pie charts showing the distribution of functional subsets of CD4^+^ and CD8^+^ T‐cells amongst the different cohorts. The percentages displayed in the graphs are calculated from the median of the absolute number of cells

In comparison to controls, patients with stable CLL (*n* = 45) had significantly lower relative numbers of all major cell populations which were due to a higher number of clonal B‐cells in the CLL patients (Table S3). Only the relative number of T‐reg was higher in the patients, as this is a percentage of CD4^+^ T‐cells and as a result, is not influenced by B‐cell count. Absolute numbers of all the major populations except for CD4^+^CD8^+^ T‐cells and polyclonal B‐cells were significantly higher in patients than in controls. As for the functional subsets, the stable patients had a significantly higher absolute number of CD4^+^ TCM, CD8^+^ TCM, and CD8^+^ TEMRA than controls and a higher percentage of CD4^+^ TCM than controls (Figure 2).

When comparing the patients with progressive disease (*n* = 86) to controls, the differences among the major cell populations observed between the stable patients and controls, were even more apparent (Table S4). Moreover, while the stable patients had more CD4^+^ TCM, CD8^+^ TCM, and CD8^+^ TEMRA than controls, the patients with progressive disease had the same changes and in addition, they also had significantly more CD4^+^ TEM, CD4^+^ TEMRA, and CD8^+^ TEM, so only naïve cell counts remained similar to that of the controls. In relative numbers, there was a significantly lower percentage of naïve cells in both the CD4^+^ and CD8^+^ compartments and a higher percentage of CD4^+^ TEM and CD8^+^ TCM in the patients (Figure 2).

Finally, we compared patients with stable (*n* = 45) and progressive disease (*n* = 80) (Table 2). The patients with progressive disease had more cells in all analyzed populations with the exception of polyclonal B‐cells, NKT, and NK cells. Because of a higher clonal B‐cell count in the patients with progressive disease, the percentages of other cells were correspondingly lower compared to the stable patients. The patients with progressive disease had significantly higher counts of CD4^+^ TCM and TEM, CD8^+^ TCM, TEM, and TEMRA than the stable patients. Relative numbers of CD4^+^ and CD8^+^ naïve cells were significantly lower, while the proportion of CD4^+^ and CD8^+^ TEM was higher in the patients with progressive disease (Figure 2). Also, the relative number of the CD8^+^ TEMRA was lower in this group, even though the absolute number was higher, because of a relatively higher increase in CD8^+^ TCM and TEM. An overview of the changes is given in Table 3.

**TABLE 2:** Comparison of patients with stable and progressive disease

Subset	Stable disease		Progressive disease		*p*‐value	
	Absolute count^a^	Relative count^a^	Absolute count^a^	Relative count^a^	Absolute count	Relative count
Total T‐cells	2.17	7.8	3.62	3.36	**<0.0001**	**0.0004**
CD4^+^ T‐cells	1.08	4.54	2.09	1.69	**<0.0001**	**0.0002**
CD8^+^ T‐cells	0.72	2.5	1.3	1.095	**0.0001**	**0.001**
CD4^+^CD8^+^ T‐cells	0.013	0.08	0.03	0.04	0.12	**0.016**
CD4^−^CD8^−^ T‐cells	0.089	0.4	0.15	0.13	**0.034**	**0.0016**
Total B‐cells	15.93	89.7	125.21	95.92	**<0.0001**	**0.0001**
Clonal B‐cells	15.82	89.56	125.1	95.78	**<0.0001**	**0.0001**
Polyclonal B‐cells	0.048	0.18	0.06	0.055	0.33	**0.0051**
NKT cells	0.15	0.5	0.17	0.18	0.46	**0.012**
NK cells	0.5	2.38	0.6	0.56	0.44	**<0.0001**
CD4^+^ naïve	0.36	35.62	0.3	15.6	0.13	**<0.0001**
CD4^+^ TCM	0.27	24.54	0.38	21.62	**0.04**	0.47
CD4^+^ TEM	0.4	*34.3*	0.92	*49.96*	**<0.0001**	**0.0001**
CD4^+^ TEMRA	0.033	2.4	0.047	2.38	0.082	0.84
CD8^+^ naïve	0.12	16.7	0.11	10.15	0.78	**0.0005**
CD8^+^ TCM	0.024	3.28	0.057	5.24	**0.0011**	0.17
CD8^+^ TEM	0.18	*27.73*	0.43	*38.58*	**<0.0001**	**<0.0001**
CD8^+^ TEMRA	0.32	*48.39*	0.48	*41.69*	**0.025**	**0.047**
T‐reg	0.084	*7.28*	0.2	*10.44*	**0.0003**	**0.0018**

Abbreviations: TCM, central memory T‐cells; TEM, effector memory T‐cells; TEMRA, terminally differentiated effector memory T‐cells.

^a^ Absolute counts are expressed in 10^9^/L, relative counts in %. Median or arithmetic means are used according to the data distribution as described in the methods. Arithmetic means are shown in italics. Statistically significant *p‐*values are in bold.

**TABLE 3:** Overview of the changes in the functional subsets of T‐cells

	CD4^+^ compartment				CD8^+^ compartment			
	Naïve	TCM	TEM	TEMRA	Naïve	TCM	TEM	TEMRA
Stable vs. controls								
Number	~	↑	~	~	~	↑	~	↑
%	~	↑	~	~	~	~	~	~
Progressive vs. controls								
Number	~	↑	↑	↑	~	↑	↑	↑
%	↓	~	↑	~	↓	↑	~	~
Progressive vs. stable								
Number	~	↑	↑	~	~	↑	↑	↑
%	↓	~	↑	~	↓	~	↑	↓
Before vs. after the treatment								
Number	↑	↑	↑	↑	↑	↑	↑	↑
%	↑	↑	↓	~	↑	↑	↓	~
Stable vs. after the treatment								
Number	↑	↑	~	↑	↑	↑	~	↑
%	↑	↑	↓	~	↑	↑	↓	~
Controls vs. after the treatment								
Number	↑	↑	~	↑	↑	~	~	~
%	↑	~	↓	~	↑	~	↓	~

*Note*: ~ symbolizes that there is no statistically significant difference between the compared groups. ↑ and ↓ symbolize that the group mentioned first in the most left column has significantly higher/lower absolute count (number), or relative count (%) of particular cells.

Abbreviations: TCM, central memory T‐cells; TEM, effector memory T‐cells; TEMRA, terminally differentiated effector memory T‐cells.

### 3.3 The effect of first‐line treatment

We analyzed the paired samples of patients before and after treatment (*n* = 55) (Table S5). The absolute numbers of all of the analyzed populations decreased significantly. The decrease in the absolute numbers of the functional subsets of T‐cells corresponded to a decrease in total T‐cells. Relative numbers of the functional subsets changed in favor of TEM cells at the expense of the naïve and TCM cells in both the CD4^+^ and CD8^+^ compartments (Figure 2). The relative number of TEMRA cells remained unchanged.

**FIGURE 3:**
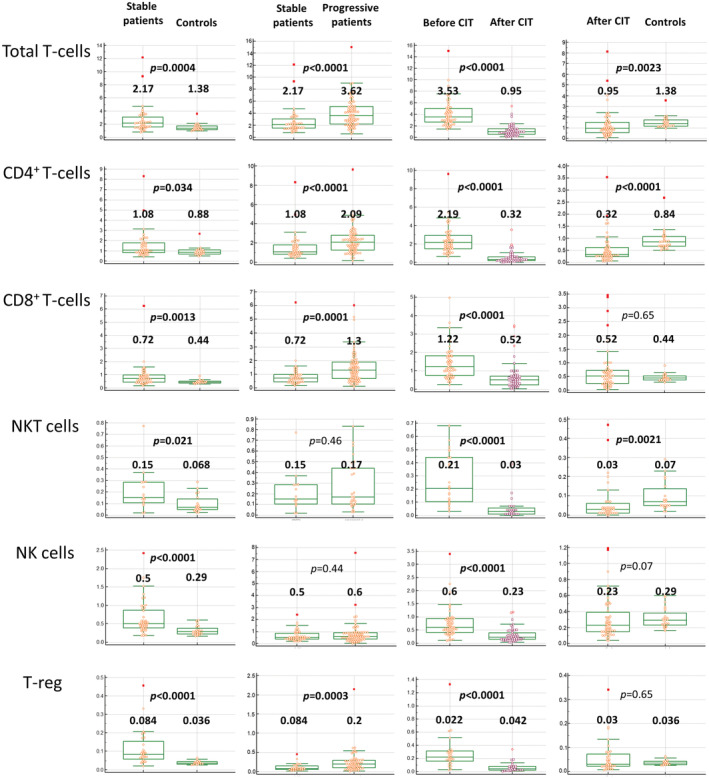
Box and whisker plots of changes in absolute numbers of the selected cell populations during disease progression and treatment. The numbers are given in 10^9^/L. The boxes represent the interquartile range and the whiskers are drawn at a distance of 1.5 times the interquartile range from the first and third quartiles.

Patients after treatment (*n* = 55) were also compared to stable patients and controls (Table S6 and Table 4). Absolute numbers of all of the analyzed populations decreased below the level seen in the stable patients with the exception of CD4^+^ and CD8^+^ TEM. Also compared to controls, who generally had lower cell counts than the stable patients, the patients after treatment had either similar or even lower numbers (significantly lower in the case of total T‐cells, CD4^+^ T‐cells, CD4^−^CD8^−^ T‐cells, polyclonal B‐cells, NKT cells, CD4^+^ naïve, TCM and TEMRA and CD8^+^ naïve cells). Despite the treatment‐related decrease in the absolute number of T‐reg that brought this cell count to a level comparable with controls, the relative number of T‐reg remained higher than in controls because other CD4^+^ cells decreased more significantly. The patients after the treatment also had a lower proportion of CD4^+^ and CD8^+^ naïve and TCM cells than the stable patients and a higher proportion of the CD4^+^ and CD8^+^ TEM than both controls and the stable patients (Figure 2). The changes in the major cell populations described above are graphically displayed in Figure 3. An overview of the changes is given in Table 3.

**TABLE 4:** Comparison of patients after treatment with the controls

Subset	After the treatment		Controls		*p*‐value	
	Absolute count^a^	Relative count^a^	Absolute count^a^	Relative count^a^	Absolute count	Relative count
Total T‐cells	0.95	74.95	1.38	73.25	**0.0023**	0.59
CD4^+^ T‐cells	0.32	26.07	0.84	44.92	**<0.0001**	**<0.0001**
CD8^+^ T‐cells	0.52	36.9	0.44	22.8	0.65	**0.0023**
CD4^+^CD8^+^ T‐cells	0.01	0.52	0.01	0.51	0.12	0.65
CD4^−^CD8^−^ T‐cells	0.01	1.11	0.05	1.99	**0.0005**	**0.0031**
Total B‐cells	0	0.05	0.23	11.25	**<0.0001**	**<0.0001**
Polyclonal B‐cells	0	0	0.23	11.25	**<0.0001**	**<0.0001**
NKT cells	0.03	1.78	0.07	3.62	**0.0021**	**0.032**
NK cells	0.23	16.12	0.29	15.012	0.07	0.39
CD4^+^ naïve	0.006	2.04	0.38	40.03	**<0.0001**	**<0.0001**
CD4^+^ TCM	0.053	14.76	0.2	16.08	**<0.0001**	0.2
CD4^+^ TEM	0.23	76.16	0.31	40.45	0.065	**<0.0001**
CD4^+^ TEMRA	0.01	3.2	0.021	1.94	**0.033**	0.53
CD8^+^ naïve	0.014	3.16	0.093	12.69	**0.0001**	**<0.0001**
CD8^+^ TCM	0.0084	1.98	0.014	2.24	0.17	0.73
CD8^+^ TEM	0.19	*43.61*	0.15	*33.46*	0.4	**0.013**
CD8^+^ TEMRA	0.19	40.29	0.22	41.62	0.93	1
T‐reg	0.03	*13.16*	0.036	*5.01*	0.65	**0.0003**

Abbreviations: TCM, central memory T‐cells; TEM, effector memory T‐cells; TEMRA, terminally differentiated effector memory T‐cells.

^a^ Absolute counts are expressed in 10^9^/L, relative counts in %. Median or arithmetic means are used according to the data distribution as described in the methods. Arithmetic means are shown in italics. Statistically significant *p‐*values are in bold.

### 3.4 A comparison of the different treatment regimens

We separately analyzed paired samples of patients who were treated with FCR (*n* = 17), BR (*n* = 18), and chlorambucil‐based regimens (*n* = 20; 9 × O‐Clb, 11 ×R‐Clb). The changes in the cell numbers were similar to the whole analyzed cohort. The analysis of the functional subsets showed that after FCR and BR treatment, the absolute number of the CD4^+^ TEMRA cells decreased significantly, while after R‐Clb/O‐Clb, this decrease was not statistically significant. Therefore, the proportion of TEMRA cells within CD4^+^ T‐cells, actually increased after R‐Clb/O‐Clb, while it remained unchanged after FCR, BR, and also when all of the patients were analyzed together. Other than that, the changes were similar across different treatment types and comparable to the cohort of all treated patients, although some of these changes did not reach statistical significance probably because of a lower number of cases.

We compared the magnitude of the changes as a result of the different treatment modalities. In this analysis, we first assessed the difference between the cell counts before and after treatment. These differences were then compared between the regimens by using the Mann–Whitney test. A decrease in total T‐cells, CD4^+^ T‐cells, CD8^+^ T‐cells, CD4^+^CD8^+^ T‐cells, CD4^−^CD8^−^ T‐cells, NKT and NK cells, and T‐reg was comparable between the regimens. As for the functional subsets, the only significant difference was that of a higher decrease in the absolute number of CD4^+^ TEMRA after FCR treatment, compared to treatment with R‐Clb/O‐Clb (median − 0.062 × 10^9^/L vs. −0.004 × 10^9^/L, *p* = 0.0062).

### 3.5 Relation to infections

We compared cell counts between stable patients who had (*n* = 22) and did not have (*n* = 23) any infections in the 3‐year period prior to sample acquisition. The infections were as followed: upper respiratory tract infection and bronchitis, *n* = 14; urinary tract infection, *n* = 2; pneumonia, *n* = 1; sepsis, *n* = 1; infectious endocarditis, *n* = 1; erysipelas, *n* = 1; herpes labialis, *n* = 1; herpes zoster, *n* = 1. The patients with infections had more clonal B‐cells (median 28.4 vs. 13.32 × 10^9^/L, *p* = 0.043), a higher relative number of clonal B‐cells (median 93.3% vs. 81.5%, *p* = 0.046), a smaller proportion of polyclonal B‐cells (median 0.14 vs. 0.24%, *p* = 0.041), NK cells (median 1.48 vs. 3.1%, *p* = 0.049), and CD4^+^ cells (median 3.2 vs. 9.29%, *p* = 0.033). The patients with infections also had lower ratios of CD4^+^ to clonal B‐cells (median 0.034 vs. 0.12, *p* = 0.039) and NK cells to clonal B‐cells as well (median 0.016 vs. 0.039, *p* = 0.04).

Regarding the patients with progressive disease who were indicated for treatment, 29 had developed infections within the 3‐year period prior to sample acquisition and 57 had not. As 10 patients had multiple infections, there were in total 46 episodes of infections involving the upper respiratory tract and bronchitis, *n* = 26; pneumonia, *n* = 6; herpes labialis, *n* = 4; phlegmon, *n* = 3; herpes zoster, *n* = 2; fever of unknown origin, *n* = 1; urinary tract infection, *n* = 1; peritonsillar abscess, *n* = 1; gastroenteritis, *n* = 1 and cytomegalovirus stomatitis, *n* = 1. The only significant differences between the two groups were a lower proportion of naïve CD4^+^ (median 11.99 vs. 18.71%, *p* = 0.041) and a higher count of CD8^+^ TEM (median 0.54 vs. 0.36 × 10^9^/L, *p* = 0.043) in patients with infections.

There were no statistically significant differences in the immunoglobulin G, A, or M levels between the patients whether they had an infection or not (data not shown). The serious (Grade ≥3) infections were not analyzed separately, because these occurred only in four and six patients in the stable and progressive cohorts respectively.

### 3.6 Prognostic significance

**FIGURE 4:**
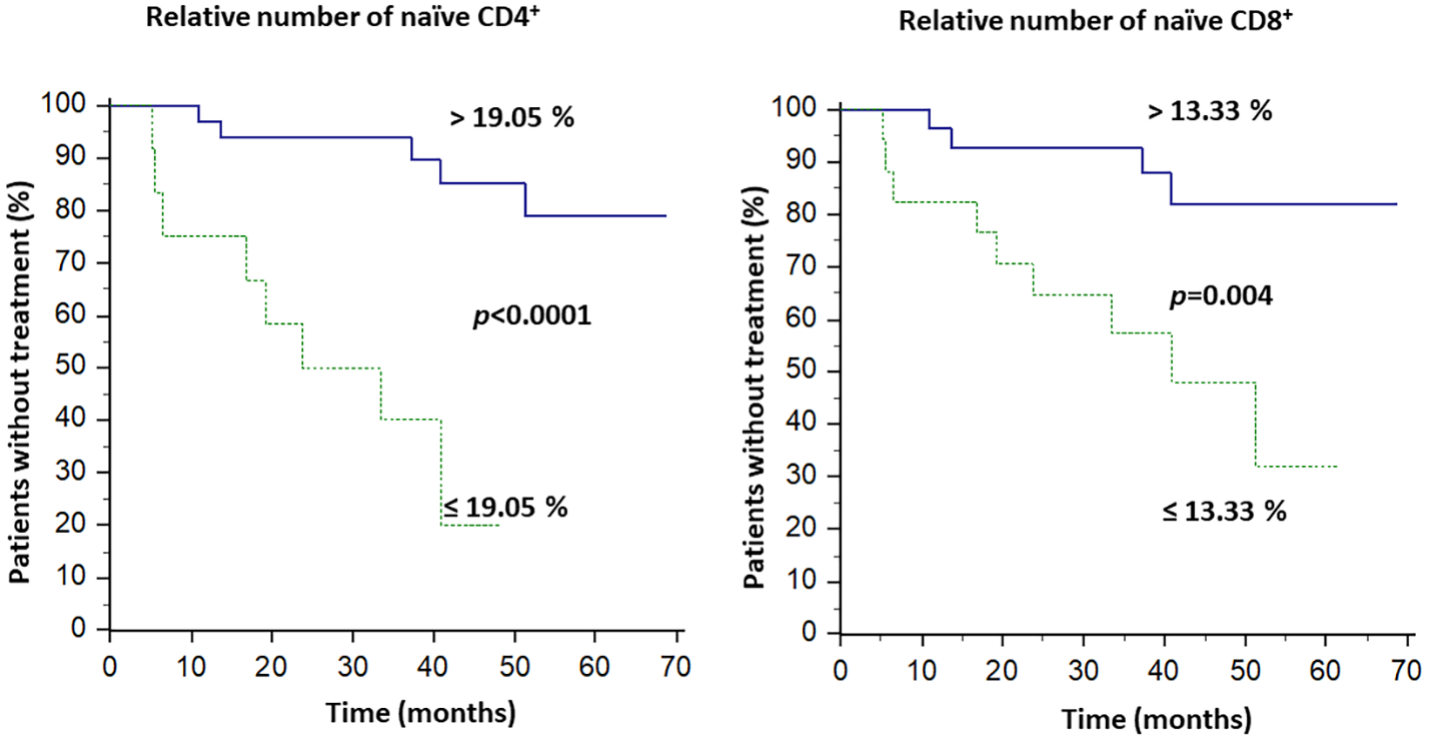
Differences between TTFT of patients based on the relative numbers of naïve CD4^+^ cells (left) and naïve CD8^+^ cells (right). ROC analysis set a cut‐off level for the best separation of curves as 19.05% for naïve CD4^+^ cells and 13.33% for naïve CD8^+^ cells.

Out of the 45 stable patients, 12 progressed to treatment and only three died during the follow‐up period. TTFT at 3 years was 79%. The higher relative number of naïve CD4^+^ (*p* = 0.0026) and naïve CD8^+^ (*p* = 0.023) was associated with longer TTFT, while the higher absolute number of CD4^+^ TCM (*p* = 0.27), relative number of CD4^+^ TEM (*p* = 0.0047), and absolute number of CD4^+^ TEM (*p* = 0.003) were associated with shorter TTFT. When analyzed together in multivariate analysis, none of these parameters retained their statistical significance. The ROC analysis was able to separate patients into two groups with significantly different TTFT in the case of the relative numbers of naïve CD4^+^ cells and naïve CD8^+^ cells (Figure 4).

No association was found between the analyzed parameters and OS in 86 patients with progressive disease (17 of them died during follow‐up; OS at 3 years was 79%).

We also analyzed the association between cell counts and OS of 56 patients who were treated with CIT. The median follow‐up of this cohort was 29 months. The higher absolute number of CD4^+^ TCM was associated with shorter OS (*p* = 0.041). However, it lost statistical significance when analyzed in the multivariate analysis.

With regard to TTNT, absolute numbers of both T‐reg (*p* = 0.042) and CD4^+^ TCM (*p* = 0.035) were associated with shorter TTNT. The CD4^+^ TCM absolute count remained significant in the multivariate analysis, independently of the treatment type and the T‐reg count (*p* = 0.024) (Table S7).

As for any association between cell counts, OS and TTNT separately for the FCR, BR, and R‐Clb/O‐Clb cohorts, in the R‐Clb/O‐Clb cohort higher absolute numbers of naïve CD4^+^ (*p* = 0.018), CD4^+^ TCM (*p* = 0.015), naïve CD8^+^ (*p* = 0.016), and CD8^+^ TCM (*p* = 0.0088) were all associated with shorter TTNT. None of these, however, retained statistical significance in the multivariate analysis.

## 4 DISCUSSION

In this present study, we performed a detailed analysis of lymphocyte subsets in patients with CLL and the changes induced by first‐line CIT.

In the studies of Nunes et al. and Wu et al., the inversion of the CD4^+^ to CD8^+^ ratio was connected to shorter TTFT, PFS, and OS.^2^, ^3^ In our analysis, the CD4^+^ to CD8^+^ ratio did not have a significant impact on TTFT of stable patients or TTNT and OS of patients with progressive disease. The difference in the results might at least partially be attributed to differences in study populations. Nunes et al. had a higher proportion of patients with the CD4^+^ to CD8^+^ ratio inversion and Wu et al. analyzed the patients with stable and progressive CLL together. Palmer et al. found that a lower ratio of total T‐cells and NK cells to clonal B‐cells is associated with shorter TTFT in patients with early CLL.^1^ Gonzalez‐Rodriguez et al., observed a significant positive correlation between CD4^+^ or CD8^+^ cells to clonal B‐cells ratios and OS.^31^ We did not observe any significant impact of measured ratios on TTFT, TTNT, or OS with one exception: a lower ratio of NKT to clonal B‐cells in progressive CLL was associated with shorter OS (*p* = 0.043). So far, only one similar finding was reported by Bojarska‐Junak et al., who observed significantly longer treatment‐free survival and OS in untreated CLL patients with NKT cells >5.3% of all T‐cells.^32^ These results suggest that NKT cells may play an important role in controlling the malignant clone.

When we analyzed absolute and relative numbers of the major cell populations, a pattern could be seen where stable CLL patients had a significantly higher absolute number of these cells than controls, and patients with progressive CLL, in turn, had higher counts than the stable ones (with the exception of CD4^+^CD8^+^ T‐cells, polyclonal B‐cells, NKT cells and NK cells). Our findings are compatible with the studies cited above, where CD4^+^ T‐cells and CD8^+^ T‐cells were also elevated above the norm or in comparison to controls.

There is a well‐established association between the prognosis and the T‐reg count. Many studies observed expansion of T‐reg number or proportion compared to healthy controls as we did.^6^, ^7^, ^8^, ^9^, ^10^, ^11^, ^12^, ^33^, ^34^ In some of them, an increase of T‐reg numbers in progressive disease was apparent as it was in our cohort.^7^, ^8^, ^33^ Several studies also reported connection between an increase in either relative or absolute number of T‐reg and shorter TTFT or OS.^10^, ^11^, ^12^ In our study, the higher absolute number of T‐reg was associated with shorter TTNT of patients treated with CIT (*p* = 0.042), but it lost its prognostic significance in the multivariate analysis. Apart from T‐reg, we did not observe any prognostic value of absolute or relative counts of any other major cell population regarding TTFT of stable patients or TTNT and OS of patients with progressive CLL. The prognostic impact of the NK cell count on OS, observed by Wang et al, was not confirmed in our study, possibly because of the smaller number of patients.^13^

Changes in the proportion of different functional subsets of CD4^+^ and CD8^+^ T‐cells were most thoroughly described by Nunes et al. and Riches et al., who focused on CD8^+^ T‐cells only.^2^, ^35^ Taking together the observations from both studies, we can summarize that while a proportion of naïve and TCM cells decrease, an increase is observed in TEM and TEMRA cells in both the CD4^+^ and CD8^+^ compartments. The expansion of CD4^+^ TEM and CD8^+^ TEM and TEMRA was connected to progressive disease.^4^, ^5^ In the study of Rissiek et al., the expansion of CD8^+^ TEM and TEMRA also had a significant negative impact on TTFT.^36^ In our work not surprisingly, the greatest differences were seen between patients with progressive CLL and controls, while the differences between stable patients and controls and stable patients and these with progressive CLL were less pronounced.

In the CD4^+^ compartment of patients with progressive disease, absolute numbers of TCM, TEM, and TEMRA cells were increased, while the naïve CD4^+^ cell count remained unchanged. Percentagewise, it led to a decline in the proportion of naïve cells and an increase in the proportion of TEM because their absolute number increased the most. Therefore, a proportion of TCM and TEMRA did not change despite the increase in their absolute counts. In the CD8^+^ compartment, there is a much higher proportion of TEMRA cells than in the CD4^+^ compartment; however, the dynamics of the changes with disease progression, were like that of CD4^+^ cells. Except for TCM cells, described changes were generally similar to the results of Nunes et al. Whereas they observed a lower percentage of CD4^+^ TCM and a trend toward it in CD8^+^ TCM in CLL patients compared to controls, in our study, the percentage of CD4^+^ TCM was higher in patients with stable disease, and the percentage of CD8^+^ TCM in patients with the progressive disease compared to controls. One possible explanation might be that our control group was not strictly age‐matched. It is well‐known that CD4^+^ TCM increases with advancing age.^16^ Another possible cause of the difference might lie in a relatively higher increase of TEM and TEMRA in the patients of Nunes et al. in comparison to our cohorts. The common observation of both our and Nunes' studies, is the decrease in the relative number of naïve cells at the expense of the more mature subsets, especially TEM and TEMRA.

The naïve cells depletion reduces the responsiveness of the immune system to new stimuli, and thus the ability to control infection or neoplastic growth. We also observed the negative impact of the depletion of CD4^+^ and CD8^+^ naïve cells on TTFT in our group of patients with stable disease. This further supports the idea of their role in tumor control. To the best of our knowledge, these findings have never been published before. Other observations in our stable patients’ group, i.e., the association of a higher absolute number of CD4^+^ TCM and TEM and a higher relative number of CD4^+^ TEM with shorter TTFT, are also novel and complement this theory. The higher absolute number of CD4^+^ TCM was also associated with shorter OS and TTNT in CIT‐treated patients and the association with TTNT remained significant in the multivariate analysis, independently of treatment type.

The evaluations of any possible association between cell counts before the treatment and OS and TTNT were also performed separately for different treatment regimens. In the R‐Clb/O‐Clb cohort, the higher absolute numbers of both CD4^+^ and CD8^+^ naïve cells and TCM cells were associated with shorter TTNT. The negative influence of the absolute count of naïve cells might be surprising, but it does not directly contradict the above‐mentioned positive impact of their higher relative numbers. As Table 3 shows, the absolute numbers of naïve cells are similar between controls and patients with stable and progressive disease. Their relative depletion is rather a result of an increase in the other subsets. To summarize this part, we conclude that the progression of CLL leads to an expansion of the TCM, TEM, and TEMRA counts in both the CD4^+^ and CD8^+^ compartments while the naïve cell counts remain stable and thus relatively deficient in their response to increased disease activity.

With regard to the changes in the lymphocyte populations induced by CIT, we detected a universal decrease in all populations that brought their numbers to levels comparable with the control group, or even lower. Though the treatment depleted all the cell types, the magnitude of the reduction was variable. A more extensive decrease was seen in CD4^+^ cells compared to CD8^+^, so the median CD4^+^ to CD8^+^ ratio deviated even further from the normal values. Although this may be considered undesirable, the CD4^+^ depletion was connected to longer PFS in patients who had detectable MRD after FCR treatment.^19^ In another study, patients with CD4^+^ counts below 0.2 × 10^9^/L after three cycles of FCR more often achieved MRD negativity.^21^ One possible explanation is that a lower reduction of the CD4^+^ population might be just mirroring a generally lower effectivity of the cytostatic treatment in a given individual. Alternatively, the residual CD4^+^ population might include T‐reg with disease‐promoting properties. In the study by Beyer et al., patients treated with fludarabine had a lower proportion of T‐reg out of all CD4^+^ cells, than patients after receiving other types of treatment and untreated ones as well.^37^ In our work, the absolute numbers of T‐reg decreased to levels comparable to controls after CIT treatment, however, the changes in other CD4^+^ cell subsets were more significant so the proportion of T‐reg actually increased.

The effect of the CIT on absolute counts of functional subsets was the same as for the major cell populations, i.e., there was a decrease to the level of the control group, or even lower. The percentage of naïve cells, which was lower in progressive disease compared to controls and stable disease, decreased even further, while TEM cells further increased. A proportion of TCM decreased and a proportion of TEMRA remained the same. These changes were similar for both CD4^+^ and CD8^+^ cells. Their relative numbers thus changed in a way that resembled the effects of disease progression, at least for naïve and TEM cells. The information on this topic is very limited throughout the literature. Both Egle et al. and Gassner et al. observed a decrease in the proportion of naïve cells after fludarabine treatment, which was similar to our own results.^21^, ^38^ While in our study, a proportion of TEM cells increased, the percentage of TCM cells decreased unlike in the research of Egle et al. Gassner et al. did not distinguish between the two subsets and demonstrated just an increase in memory cells. These studies used methods that were different from ours (Gassner's work was done mostly on cells exposed to chemotherapy ex vivo and different staining methods were used for flow cytometry); hence, some differences are to be expected. A result that we had in common with the other studies was a decrease in the percentage of naïve cells at the expense of more mature subsets in the same manner as we and the others saw as a consequence of disease progression. This again might be detrimental to disease control and the patients' resistance to infections.

Chlorambucil‐based regimens caused a similar decrease in the lymphocyte counts as did FCR and BR. The only difference from FCR was that the decrease in the absolute count of CD4^+^ TEMRA after R‐Clb/O‐Clb did not reach statistical significance and accordingly, an increase in their relative numbers did, which was not the case for FCR/BR. The reason for this became apparent when we compared the magnitude of the changes caused by different types of treatment. The decrease in CD4^+^ TEMRA after R‐Clb/O‐Clb was much smaller than after FCR, which was the only significant difference between these two regimens.

The effects of all three types of treatment were remarkably similar. This might be surprising, especially for chlorambucil‐based treatment. Its' effects were previously described in only two studies: Laszlo et al. described the depletion of CD4^+^ T‐cells in 25 patients with indolent lymphomas who were treated with chlorambucil and rituximab.^26^ In recently published data from the RESONATE‐2 study, all major lymphocyte populations were reduced after chlorambucil monotherapy.^27^ We provide further evidence that chlorambucil‐based treatment may cause lymphodepletion just like bendamustine and fludarabine. It is also necessary to consider that the anti‐CD20 antibody is partially responsible for changes in cell counts. In the study of lymphocyte populations of patients with rheumatoid arthritis who were treated with rituximab monotherapy, 75% of them had a decrease in the CD4^+^ cell count by at least 21%.^39^ The reason for this might be a depletion of B‐cells and their cytokines and/or antigen‐presenting functions. It is necessary to mention the weaknesses of this subanalysis. Apart from the low number of patients treated with different chemotherapy regimens, it is also true that the patients in these groups differed significantly in their age and comorbidities.

Regarding the association of lymphocyte subsets with infectious complications, we separately assessed patients with stable and progressive disease. Amongst the stable patients, we observed more infections in those with more clonal B‐cells, a higher proportion of clonal B‐cells out of all B‐cells, a higher proportion of NK cells out of all lymphocytes (including clonal B‐cells), and in those with lower CD4^+^ to clonal B‐cells and NK cells to clonal B‐cells ratios. All of these results indicate the importance of tumor burden rather than that of the total CD4^+^ or NK cell counts because their absolute numbers were not connected to infections in a statistically significant manner; more advanced disease is naturally connected to a higher level of immunosuppression in all parts of the immune system. More interesting results were apparent when we analyzed the infections in the patients with progressive disease. Those who developed infections had a lower percentage of naïve CD4^+^ cells and a higher absolute number of CD8^+^ TEM. This may lead to the conclusion that a relative deficiency in naïve cells and the expansion of more mature subsets in progressive CLL might render these patients more susceptible to infections. On the contrary, it is equally possible that infections are just more common in patients with more advanced diseases, which also features a depletion of naïve cells at the expense of TEM as we have shown. The infections then might instead be connected to some other variables in immunity, which are related to progressive disease, and not just to changes in the lymphocyte counts.

## 5 CONCLUSIONS

We performed a complex assessment of the changes in lymphocyte populations in patients with stable as well as progressive CLL. In addition, the effects of first‐line treatment with different types of CIT were investigated. We observed increases in most of the lymphocyte subsets including T‐reg and a shift from naïve T‐cells to more mature cells (TCM, TEM, TEMRA) in CLL, compared to controls and also with those with progressive CLL. We provide hitherto unpublished evidence of the negative prognostic impact of the depletion of CD4^+^ and CD8^+^ naïve cells and the increase of CD4^+^ TCM and CD4^+^ TEM. Furthermore, we bring evidence that a lower percentage of naïve CD4^+^ cells and a higher absolute number of CD8^+^ TEM, are associated with a higher risk of infectious complications. To the best of our knowledge, our study is also the first to report changes in the different functional subsets of the T‐cells after BR treatment and complements the scarce data available about such changes after FCR and chlorambucil‐based therapy. The possible weaknesses of our study include its non‐randomized design when comparing different treatment regimens, relatively low number of patients, and its unicentric design. Further studies in larger patient cohorts are warranted to confirm our results.

## AUTHOR CONTRIBUTIONS

**Pavel Vodárek:** Conceptualization (equal); data curation (equal); formal analysis (equal); funding acquisition (equal); investigation (equal); methodology (equal); project administration (lead); writing – original draft (lead); writing – review and editing (lead). **Dominika Écsiová:** Conceptualization (equal); data curation (equal); formal analysis (equal); funding acquisition (equal); investigation (equal); methodology (equal); project administration (supporting); writing – review and editing (supporting). **Vladimira Řezáčová:** Conceptualization (equal); data curation (equal); formal analysis (equal); funding acquisition (equal); investigation (equal); methodology (equal); project administration (supporting); writing – review and editing (supporting). **Ondřej Souček:** Conceptualization (equal); data curation (equal); formal analysis (equal); funding acquisition (equal); investigation (equal); methodology (equal); project administration (supporting); writing – review and editing (supporting). **Martin Šimkovič:** Conceptualization (equal); data curation (equal); formal analysis (equal); funding acquisition (equal); investigation (equal); methodology (equal); project administration (supporting); writing – review and editing (supporting). **Doris Vokurková:** Conceptualization (equal); data curation (equal); formal analysis (equal); funding acquisition (equal); investigation (equal); methodology (equal); project administration (supporting); writing – review and editing (supporting). **David Belada:** Conceptualization (equal); data curation (equal); formal analysis (equal); funding acquisition (equal); investigation (equal); methodology (equal); project administration (supporting); supervision (equal); writing – review and editing (supporting). **Pavel Žák:** Conceptualization (equal); data curation (equal); formal analysis (equal); funding acquisition (equal); investigation (equal); methodology (equal); project administration (supporting); writing – review and editing (supporting). **Lukáš Smolej:** Conceptualization (equal); data curation (equal); formal analysis (equal); funding acquisition (equal); investigation (equal); methodology (equal); project administration (supporting); supervision (equal); writing – review and editing (supporting).

## FUNDING STATEMENT

This work was supported by MH CZ – DRO (UHHK, 00179906) from the Ministry of Health, Czech Republic, by program Cooperation, research area ONCO, by the League Against Cancer Prague, and by Charles University in Prague, Faculty of Medicine in Hradec Kralove's program for postdoctoral students.

## CONFLICT OF INTEREST

Pavel Vodárek reports consultations for Roche, Gilead, Janssen‐Cilag and Servier, research funding from Roche, honoraria and travel grants from AbbVie, Roche, Gilead, Janssen‐Cilag, Servier, and Celgene. Martin Šimkovič reports consultancy fees, advisory board participation fees, travel grants, and honoraria from Janssen‐Cilag, Gilead, Roche, AstraZeneca, and AbbVie. David Belada reports consultancy fees, advisory board participation fees, travel grants and honoraria from Roche, Takeda, Gilead, and Janssen Cilag. Lukáš Smolej reports honoraria, consultancy fees and travel grants from Roche, AbbVie, Janssen, and AstraZeneca. Other authors report no conflicts of interest.

## ETHICS APPROVAL AND PATIENT CONSENT STATEMENT

All patients signed the necessary informed consents, the study was approved by the local ethics committee and conducted according to the principles of the Declaration of Helsinki.

## Supporting information


Table S1.

## Data Availability

The data that support the findings of this study are available on request from the corresponding author. The data are not publicly available due to privacy or ethical restrictions.
