# Supplementary material for: A comprehensive assessment of lymphocyte subsets, their prognostic significance, and changes after first‐line therapy administration in patients with chronic lymphocytic leukemia
Source: Cancer Med. 2022 Nov 28;12(6):6956–70. doi: 10.1002/cam4.5492 (PMC10067047; doi:10.1002/cam4.5492)
Supplement: Supplementary file 1 — Table S1. [file CAM4-12-6956-s001.docx]

**Supplementary Appendix**

1. Staining and procedures used in flow cytometry analysis.

For the surface staining of leukocytes, 50 µl of anticoagulated blood was added to tubes containing 5 µl of fluorochrome-labelled monoclonal antibodies, including anti-CD3 Phycoerythrin-Texas Red-x (ECD), clone UCHT1; anti-CD4 Krome Orange (KrO), clone 13B8.2; anti-CD19 phycoerythrin-cyanin 7 (PC7), clone J3-119; anti-CD20 Pacific Blue (PB), clone B9E9; anti-CD5 Phycoerythrin-cyanin 5.5 (PC5.5), clone BL1a; anti-kappa chain Fluorescein Isothiocyanate (FITC), isotype F(ab´)2 Goat ; anti-lambda chain Phycoerythrin (PE), isotype F(ab´)2 Goat; anti-CD8 Fluorescein Isothiocyanate (FITC), clone B9.11; anti-CD8 Allophycocyanin (APC), clone B9.11; anti-CD16 Phycoerythrin (PE), clone 3G8; anti-CD25 PE, clone B1.49.9; anti-CD27 allophycocyanin-Alexa Fluor 750 (APC-A750), clone 1A4CD27; anti-CD28 Phycoerythrin-cyanin 5.5 (PC5.5), clone CD28.2; anti-CD45 KrO, clone J33; anti-CD45RA phycoerythrin-cyanin 7 (PC7), clone 2H4LDH11LDB9; anti-CD45RO FITC; clone UCHL1; anti-CD56APC, clone N901 (NKH-1); anti-CD57 Pacific Blue (PB), clone NC1; anti-CD127 PC7, clone R34.34; anti-CD197 (CCR7) PE, clone G043H7; anti-FoxP3 Alexa Fluor 488 (AF488); clone PCH101. The anti-FoxP3 AF488 antibody was manufactured by eBioscience (San Diego, CA, USA) and all of the other antibodies were manufactured by Beckman Coulter (Miami, FL, USA). The blood samples were incubated for fifteen minutes with antibodies at room temperature in the dark. Then, a lysing solution (OptiLyse C, Beckman Coulter) was added, and the mixture was incubated for another 10 minutes. The flow cytometric evaluation was conducted with Navios 10 flow cytometer (Beckman Coulter). All of the data was then assessed by using the Kaluza 2.1 Analysis Software (Beckman Coulter). The data at a minimum of 10,000 CD3^+^ events was obtained for each staining and supplied as a list mode. For FoxP3 intracellular staining, the eBioscience™ Human Regulatory T Cell Staining Kit (eBioscience) was used according to the manufacturer´s instruction.

2. Supplementary Table 1. Table with the characteristics of the different treatment cohorts. The data correspond to the pre-treatment values.

| **Characteristic** | FCR | BR | R-Clb / O-Clb |
| --- | --- | --- | --- |
| Total number | 17 | 18 | 20 |
| Age, median (range)  at the date of blood collection | 62 years  (43-67) | 67 years  (54-72) | 76 years  (64-87) |
| Males | 12 (71%) | 10 (56%) | 10 (50%) |
| Median follow-up  from the date of blood collection | 24 months | 45 months | 28 months |
| Median time (range) from diagnosis  to the date of blood collection | 41 months  (4-172) | 17 months  (1-110) | 54 months  (0-174) |
| Rai modified risk  at the date of blood collection |  |  |  |
| - low | 0 | 0 | 1 (5%) |
| - intermediate | 10 (59%) | 7 (39%) | 5 (25%) |
| - high | 7 (41%) | 11 (61%) | 14 (70%) |
| IGHV |  |  |  |
| - mutated | 1 (6%) | 3 (17%) | 7 (35%) |
| - unmutated | 14 (82%) | 12 (67%) | 8 (40%) |
| - NA | 2 (12%) | 3 (17%) | 5 (25%) |
| *TP53* |  |  |  |
| - mutated | 0 | 1 (6%) | 4 (20%) |
| - unmutated | 13 (76%) | 13 (72%) | 13 (65%) |
| - NA | 4 (24%) | 4 (22%) | 3 (15%) |
| FISH |  |  |  |
| - normal | 5 (30%) | 5 (28%) | 5 (25%) |
| - 13q deletion | 3 (18%) | 8 (44%) | 5 (25%) |
| - 12 trisomy | 3 (18%) | 2 (11%) | 3 (15%) |
| - 11q deletion | 5 (30%) | 2 (11%) | 1 (5%) |
| - 17p deletion | 0 | 1 (6%) | 1 (5%) |
| - NA | 1 (6%) | 0 | 4 (20%) |
| *TP53* mutation or 17p deletion | 0 | 2 (11%) | 4 (20%) |

Rai modified risk: stage 0 = low, stage I/II = intermediate, stage III/IV = high. IGHV – mutational status of the immunoglobulin heavy chain variable region. FISH – cytogenetic aberrations detected by fluorescence in situ hybridization. *TP53* – mutation of tumour protein 53 investigated by Sanger sequencing. NA – not available. FCR – fludarabine, cyclophosphamide, rituximab. BR – bendamustine, rituximab. O-Clb – obinutuzumab, chlorambucil. R-Clb – rituximab, chlorambucil.

3. Supplementary Table 2. Comparison of the different cell count ratios between the patients with stable and progressive disease

| Ratio | Stable disease (n=45)  median (range) | Progressive disease (n=86) median (range) | *p* value |
| --- | --- | --- | --- |
| CD4^+^ : CD8^+^ | 1.85 (0.65-8,64) | 1.67 (0.11-7.64) | 0.64 |
| CD4^+^ : clonal B-cells | 0.052 (0.007-0.68) | 0.018 (0.004-75.08) | **0.0001** |
| CD8^+^ : clonal B-cells | 0.03 (0.003-0.29) | 0.012 (0.001-32.38) | **0.0008** |
| T-cells : clonal B-cells | 0.086 (0.017-1.05) | 0.035 (0.005-114.15) | **0.0003** |
| NK cells : clonal B-cells | 0.029 (0.0013-0.2) | 0.006 (0-49.17) | **<0.0001** |
| NKT cells : clonal B-cells † | 0.005 (0.001-0.19) | 0.002 (0-0.069) | **0.022** |

†NKT cells were measured only in 15 patients with stable disease and 34 patients with progressive disease

4. Supplementary Table 3. Comparison of patients with stable disease and the controls.

| Subset | Stable disease | | Controls | | *p* value | |
| --- | --- | --- | --- | --- | --- | --- |
|  | Absolute count† | Relative count† | Absolute count† | Relative count† | Absolute count | Relative  count |
| Total T-cells | 2.17 | 7.8 | 1.38 | 73.25 | **0.0004** | **<0.0001** |
| CD4^+^ T-cells | 1.08 | 4.54 | 0.88 | 46.28 | **0.034** | **<0.0001** |
| CD8^+^ T-cells | 0.72 | 2.5 | 0.44 | 21.96 | **0.0013** | **<0.0001** |
| CD4^+^CD8^+^ T-cells | 0.013 | 0.08 | 0.0095 | 0.51 | 0.12 | **<0.0001** |
| CD4^-^CD8^-^ T-cells | 0.089 | 0.4 | 0.046 | 1.99 | **0.0034** | **<0.0001** |
| Total B-cells | 15.9 | 89.7 | 0.23 | 11.25 | **<0.0001** | **<0.0001** |
| Polyclonal B-cells | 0.048 | 0.18 | 0.23 | 11.25 | **<0.0001** | **<0.0001** |
| NKT cells | 0.15 | 0.5 | 0.068 | 3.62 | **0.021** | **<0.0001** |
| NK cells | 0.5 | 2.38 | 0.29 | 15.02 | **<0.0001** | **<0.0001** |
| CD4^+^ naïve | 0.36 | *35.43* | 0.38 | *38.87* | 0.88 | 0.46 |
| CD4^+^ TCM | 0.27 | 25.98 | 0.2 | 18.09 | **0.034** | **0.013** |
| CD4^+^ TEM | 0.4 | *34.3* | 0.31 | *39.78* | 0.62 | 0.24 |
| CD4^+^ TEMRA | 0.033 | 2.4 | 0.021 | 1.94 | 0.42 | 0.86 |
| CD8^+^ naïve | 0.12 | 16.7 | 0.093 | 12.69 | 0.3 | 0.69 |
| CD8^+^ TCM | 0.024 | 3.28 | 0.014 | 2.24 | **0.027** | 0.053 |
| CD8^+^ TEM | 0.18 | *27.73* | 0.15 | *33.46* | 0.2 | 0.08 |
| CD8^+^ TEMRA | 0.32 | *48.39* | 0.22 | *43.67* | **0.034** | 0.34 |
| T-reg | 0.084 | *7.28* | 0.036 | *5.09* | **<0.0001** | **0.0031** |

† Absolute counts are expressed in 10^9^/L, relative counts in %. Median or arithmetic means are used according to the data distribution as described in the methods. Arithmetic means are shown in italics. Statistically significant *p* values are in bold. TCM – central memory T-cells. TEM – effector memory T-cells. TEMRA – terminally differentiated effector memory T-cells.

5. Supplementary Table 4. Comparison of patients with progressive disease and the controls.

| Subset | Progressive disease | | Controls | | *p* value | |
| --- | --- | --- | --- | --- | --- | --- |
|  | Absolute count† | Relative count† | Absolute count† | Relative count† | Absolute count | Relative  count |
| Total T-cells | 3.53 | 3.36 | 1.38 | 73.25 | **<0.0001** | **<0.0001** |
| CD4^+^ T-cells | 2 | 1.69 | 0.84 | 44.92 | **<0.0001** | **<0.0001** |
| CD8^+^ T-cells | 1.27 | 1.13 | 0.44 | 21.96 | **<0.0001** | **<0.0001** |
| CD4^+^CD8^+^ T-cells | 0.03 | 0.04 | 0.01 | 0.51 | **0.011** | **<0.0001** |
| CD4^-^CD8^-^ T-cells | 0.15 | 0.14 | 0.05 | 1.99 | **0.0001** | **<0.0001** |
| Total B-cells | 125.21 | 95.92 | 0.23 | 11.25 | **<0.0001** | **<0.0001** |
| Polyclonal B-cells | 0.06 | 0.06 | 0.23 | 11.25 | **<0.0001** | **<0.0001** |
| NKT cells | 0.17 | 0.19 | 0.07 | 3.62 | **0.0007** | **0.0003** |
| NK cells | 0.6 | 0.6 | 0.29 | 15.02 | **<0.0001** | **<0.0001** |
| CD4^+^ naïve | 0.28 | 15.5 | 0.38 | 40.03 | 0.3 | **<0.0001** |
| CD4^+^ TCM | 0.38 | 21.46 | 0.2 | 16.08 | **0.0037** | 0.2 |
| CD4^+^ TEM | 0.96 | *51.3* | 0.31 | *39.78* | **0.0001** | **0.03** |
| CD4^+^ TEMRA | 0.047 | 2.38 | 0.021 | 1.94 | **0.039** | 0.78 |
| CD8^+^ naïve | 0.11 | 9.92 | 0.093 | 12.69 | 0.32 | **0.046** |
| CD8^+^ TCM | 0.056 | 5.22 | 0.014 | 2.24 | **0.0002** | **0.0036** |
| CD8^+^ TEM | 0.44 | *38.95* | 0.15 | *33.46* | **<0.0001** | 0.14 |
| CD8^+^ TEMRA | 0.48 | 41.83 | 0.22 | 43.67 | **0.0012** | 0.69 |
| T-reg | 0.19 | *10.44* | 0.036 | *5.01* | **<0.0001** | **0.0001** |

† Absolute counts are expressed in 10^9^/L, relative counts in %. Median or arithmetic means are used according to the data distribution as described in the methods. Arithmetic means are shown in italics. Statistically significant *p* values are in bold. TCM – central memory T-cells. TEM – effector memory T-cells. TEMRA – terminally differentiated effector memory T-cells.

6. Supplementary Table 5. Comparison of patients before and after treatment (paired samples).

| Subset | Before the treatment | | After the treatment | | *p* value | |
| --- | --- | --- | --- | --- | --- | --- |
|  | Absolute count† | Relative count† | Absolute count† | Relative count† | Absolute count | Relative  count |
| Total T-cells | 3.53 | 2.57 | 0.95 | 74.95 | **<0.0001** | **<0.0001** |
| CD4^+^ T-cells | 2.19 | 1.56 | 0.32 | 26.07 | **<0.0001** | **<0.0001** |
| CD8^+^ T-cells | 1.22 | 0.85 | 0.52 | 38.72 | **<0.0001** | **<0.0001** |
| CD4^+^CD8^+^ T-cells | 0.03 | 0.03 | 0.01 | 0.52 | **<0.0001** | **<0.0001** |
| CD4^-^CD8^-^ T-cells | 0.15 | 0.12 | 0.01 | 1.11 | **<0.0001** | **<0.0001** |
| Total B-cells | 175.34 | 96.77 | 0 | 0.05 | **<0.0001** | **<0.0001** |
| Clonal B-cells | 165.94 | 96.71 | 0 | 0 | **<0.0001** | **<0.0001** |
| Polyclonal B-cells | 0.06 | 0.05 | 0 | 0 | **<0.0001** | **0.0003** |
| NKT cells | 0.21 | 0.17 | 0.03 | 1.92 | **<0.0001** | **<0.0001** |
| NK cells | 0.6 | 0.48 | 0.23 | 16.1 | **<0.0001** | **<0.0001** |
| CD4^+^ naïve | 0.27 | 15.15 | 0.0064 | 2.14 | **<0.0001** | **<0.0001** |
| CD4^+^ TCM | 0.44 | 20.15 | 0.054 | 14.79 | **<0.0001** | **0.0015** |
| CD4^+^ TEM | 1.05 | 53.18 | 0.24 | 75.84 | **<0.0001** | **<0.0001** |
| CD4^+^ TEMRA | 0.045 | 2.24 | 0.01 | 3.045 | **<0.0001** | 0.18 |
| CD8^+^ naïve | 0.11 | 10.15 | 0.015 | 3.19 | **<0.0001** | **0.0001** |
| CD8^+^ TCM | 0.06 | 4.3 | 0.0085 | 2.01 | **<0.0001** | **0.0005** |
| CD8^+^ TEM | 0.44 | *38.55* | 0.19 | *44.38* | **0.0002** | **0.0081** |
| CD8^+^ TEMRA | 0.49 | 40.57 | 0.19 | 39.45 | **<0.0001** | 0.16 |
| T-reg | 0.22 | *11.13* | 0.042 | *14.98* | **<0.0001** | **0.047** |

† Absolute counts are expressed in 10^9^/L, relative counts in %. Median or arithmetic means are used according to the data distribution as described in the methods. Arithmetic means are shown in italics. Statistically significant *p* values are in bold. TCM – central memory T-cells. TEM – effector memory T-cells. TEMRA – terminally differentiated effector memory T-cells.

7. Supplementary Table 6. Comparison of patients after treatment with patients with stable disease.

| Subset | Stable patients | | After the treatment | | *p* value | |
| --- | --- | --- | --- | --- | --- | --- |
|  | Absolute count† | Relative count† | Absolute count† | Relative count† | Absolute count | Relative  count |
| Total T-cells | 2.17 | 7.8 | 0.95 | 75.21 | **<0.0001** | **<0.0001** |
| CD4^+^ T-cells | 1.08 | 4.54 | 0.34 | 26.07 | **<0.0001** | **<0.0001** |
| CD8^+^ T-cells | 0.72 | 2.5 | 0.52 | 37.99 | **0.016** | **<0.0001** |
| CD4^+^CD8^+^ T-cells | 0.013 | 0.08 | 0.01 | 0.45 | **0.0008** | **<0.0001** |
| CD4^-^CD8^-^ T-cells | 0.089 | 0.4 | 0.01 | 1.11 | **<0.0001** | **<0.0001** |
| Total B-cells | 15.93 | 89.7 | 0 | 0.05 | **<0.0001** | **<0.0001** |
| Clonal B-cells | 15.82 | 89.56 | 0 | 0 | **<0.0001** | **<0.0001** |
| Polyclonal B-cells | 0.048 | 0.18 | 0 | 0 | **<0.0001** | **<0.0001** |
| NKT cells | 0.15 | 0.5 | 0.03 | 1.77 | **0.0009** | **0.0013** |
| NK cells | 0.5 | 2.38 | 0.23 | 15.97 | **<0.0001** | **<0.0001** |
| CD4^+^ naïve | 0.36 | 35.62 | 0.007 | 2.43 | **<0.0001** | **<0.0001** |
| CD4^+^ TCM | 0.27 | 24.54 | 0.055 | 13.83 | **0.0001** | **<0.0001** |
| CD4^+^ TEM | 0.4 | 32.54 | 0.23 | 76.64 | 0.068 | **<0.0001** |
| CD4^+^ TEMRA | 0.033 | 2.4 | 0.012 | 3.69 | **0.0023** | 0.13 |
| CD8^+^ naïve | 0.12 | 16.7 | 0.016 | 3.16 | **<0.0001** | **<0.0001** |
| CD8^+^ TCM | 0.024 | 3.28 | 0.0084 | 1.61 | **0.0009** | **0.026** |
| CD8^+^ TEM | 0.18 | *27.73* | 0.19 | *44.73* | 0.98 | **<0.0001** |
| CD8^+^ TEMRA | 0.32 | 48.8 | 0.19 | 40.29 | **0.033** | 0.24 |
| T-reg | 0.084 | *7.28* | 0.037 | *13.35* | **0.0006** | **0.0004** |

† Absolute counts are expressed in 10^9^/L, relative counts in %. Median or arithmetic means are used according to the data distribution as described in the methods. Arithmetic means are shown in italics. Statistically significant *p* values are in bold. TCM – central memory T-cells. TEM – effector memory T-cells. TEMRA – terminally differentiated effector memory T-cells.

8. Supplementary Table 7. Multivariate analysis of TTNT of patients treated with CIT according to the Cox regression model.

|  | Hazard ratio | 95% confidence interval | *p* value |
| --- | --- | --- | --- |
| CD4^+^ TCM absolute count | 11,06 | 1,37 to 89,23 | **0,024** |
| T-reg absolute count | 0,37 | 0,045 to 3,02 | 0,35 |
| FCR vs. BR | 0,39 | 0,072 to 2,15 | 0,28 |
| FCR vs. O-Clb | 0,90 | 0,14 to 5,61 | 0,91 |
| FCR vs. R-Clb | 1,75 | 0,31 to 9,97 | 0,53 |

TCM – central memory T-cells. FCR – fludarabine, cyclophosphamide, rituximab. BR – bendamustine, rituximab. O-Clb – obinutuzumab, chlorambucil. R-Clb – rituximab, chlorambucil.
